# Mycotoxin Contamination in Hazelnut: Current Status, Analytical Strategies, and Future Prospects

**DOI:** 10.3390/toxins15020099

**Published:** 2023-01-19

**Authors:** Maria Michela Salvatore, Anna Andolfi, Rosario Nicoletti

**Affiliations:** 1 Department of Chemical Sciences, University of Naples Federico II, 80126 Naples, Italy; 2 Institute for Sustainable Plant Protection, National Research Council, 80055 Portici, Italy; 3 BAT Center—Interuniversity Center for Studies on Bioinspired Agro-Environmental Technology, University of Naples Federico II, 80055 Portici, Italy; 4 Department of Agricultural Sciences, University of Naples Federico II, 80055 Portici, Italy; 5 Council for Agricultural Research and Economics, Research Center for Olive, Fruit, and Citrus Crops, 81100 Caserta, Italy

**Keywords:** fungal secondary metabolites, analytical techniques, detoxification, decontamination, *Corylus avellanae*

## Abstract

Hazelnuts represent a potential source of mycotoxins that pose a public health issue due to their increasing consumption as food ingredients worldwide. Hazelnuts contamination by mycotoxins may derive from fungal infections occurring during fruit development, or in postharvest. The present review considers the available data on mycotoxins detected in hazelnuts, on fungal species reported as infecting hazelnut fruit, and general analytical approaches adopted for mycotoxin investigation. Prompted by the European safety regulation concerning hazelnuts, many analytical methods have focused on the determination of levels of aflatoxin B1 (AFB1) and total aflatoxins. An overview of the available data shows that a multiplicity of fungal species and further mycotoxins have been detected in hazelnuts, including anthraquinones, cyclodepsipeptides, ochratoxins, sterigmatocystins, trichothecenes, and more. Hence, the importance is highlighted in developing suitable methods for the concurrent detection of a broad spectrum of these mycotoxins. Moreover, control strategies to be employed before and after harvest in the aim of controlling the fungal contamination, and in reducing or inactivating mycotoxins in hazelnuts, are discussed.

## 1. Introduction

Hazelnut is one of the most commonly cultivated nut crops worldwide, with Turkey (665,000 tons) and Italy (140,560 tons) representing the leading countries in the global production, with a market portion in constant growth [1]. In fact, hazelnut kernels are a key ingredient for bakery, confectionary, and chocolate products, due to their characteristic flavor and good nutritional properties [2]. The qualitative composition characterized by a special assortment of fats, proteins, carbohydrates, fiber, and vitamins qualifies the nutritional properties of hazelnuts, and accounts for their beneficial effects on health [3].

The abundance of nutrients in hazelnuts, such as lipids and carbohydrates, makes them susceptible to decay and to the development of pathogenic and saprophytic fungi that are of utmost concern for producing mycotoxins, which are known for their cytotoxic, mutagenic, neurotoxic, and carcinogenic effects in humans and animals [4]. Exposure to mycotoxins can happen by eating contaminated foods or from animals that are fed contaminated feed. These fungal secondary metabolites are produced in the field and/or during storage, when environmental conditions are favorable for fungal growth [5], and are very difficult to eliminate from the food chain, causing a loss of product, and economic damage [6].

In this context, mycotoxin control in hazelnuts is of greatest importance, and is a global challenge to safeguard consumers’ health. Nevertheless, to date, only aflatoxin B1 (AFB1) and total aflatoxins have been included in the European safety regulation concerning hazelnuts [7].

In this review, we compile the available data on mycotoxins detected in hazelnuts, and on fungal species reported as infecting hazelnut fruit. We intend to generate interest among researchers and stakeholders to investigate the multiplicity of mycotoxins, without focusing on a single or target group of mycotoxins (e.g., aflatoxins). Furthermore, we also discuss some aspects concerning control strategies to be employed before and after harvest, to reduce or to inactivate mycotoxins in hazelnuts.

## 2. Occurrence of Mycotoxins in Hazelnuts

Mycotoxins identified in hazelnuts have a great diversity in chemical structure belonging to different classes of natural products, including aflatoxins, amino acid derivatives, anthraquinones, benzodiazepines, cyclodepsipeptides, macrolides, ochratoxins, resorcylic acid lactones, sterigmatocystins, trichothecenes, and several miscellaneous compounds (Table 1). This structural heterogeneity reflects a huge variety of toxic effects, with an impact on health essentially depending on the consumed amount and their occurrence in varied assortments.

**Figure 1:**
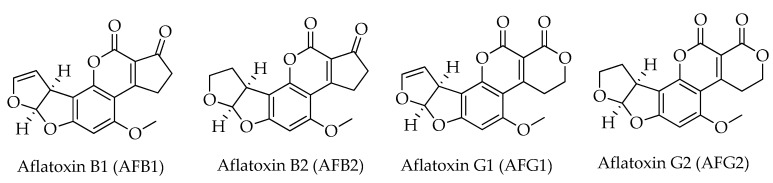
Chemical structures of aflatoxins (AFs) detected in hazelnuts.

On a worldwide scale, aflatoxins represent the most important mycotoxins in food and animal feedstuffs, raising the greatest concern due to their frequent occurrence and severe effects on health [23,24]. Aflatoxin B1 (AFB1) is classified as a group 1 human carcinogen by the International Agency for Research on Cancer (IARC) [25]. The European Commission has laid down maximum levels for AFB1 and total aflatoxins (i.e., the sum of aflatoxins B1, B2, G1, and G2) in hazelnuts for direct human consumption and/or for use as an ingredient in foodstuffs, which are 5 µg/kg for AFB1 and 10 µg/kg for total aflatoxins [7]. From a chemical perspective, aflatoxins are highly substituted coumarins: AFB1 and AFB2 have a difuro-coumaro-cyclopentenone structure, while a five-membered lactone ring replaces the cyclopentenone in AFG1 and AFG2 (Figure 1).

*Aspergillus* spp. in the section *Flavi* are the most widespread aflatoxin producers [23]. The presence of these mycotoxins in hazelnuts has been investigated in many countries, such as Turkey [11,12,15,17,26], Italy [8], China [16], Iraq [10], Bosnia-Herzegovina [18], Germany [13], and Portugal [19]. Following the increasing global trade of food products, the European Commission has recently implemented border controls on aflatoxins in nuts, which have proven to be relevant for reducing the health risk for population [27]. As an example, a study by Imperato et al. [9] revealed a high rate of contamination for hazelnuts and foods containing hazelnuts, imported in Italy from Turkey. Demirhan et al. [28] investigated the mycotoxin contamination of nut-based products (e.g., hazelnut butter and chocolate), obtained from local markets in Ankara (Turkey), observing that most samples were contaminated by AFB1 and other mycotoxins.

**Figure 2:**
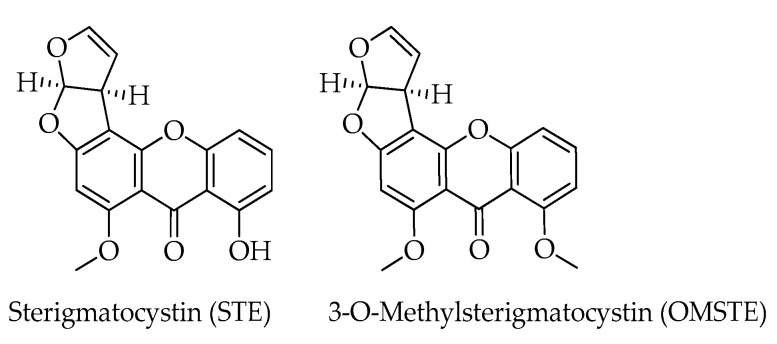
Chemical structures of sterigmatocystins detected in hazelnuts.

Sterigmatocystin (STE) and its 3-*O*-methyl derivative (OMSTE) have been also identified in hazelnuts [12]. STE was isolated for the first time from cultures of *Aspergillus versicolor*, but subsequently, species from different fungal genera (e.g., *Aschersonia*, *Botryotrichum*, *Fusarium*) showed the ability to produce this secondary metabolite [29]. STEs consist of a xanthone nucleus bond to a bifuranic structure with a hydroxyl or a methoxy group (Figure 2). STE is biosynthesized through the acetate-malonate pathway, and can be converted to OMSTE, and then to aflatoxins. In fact, the oxidative cleavage of the aromatic ring with the loss of one carbon and recyclization generates both AFB1 and AFG2. As a biosynthetic precursors of aflatoxins, it is not unusual to find these mycotoxins in the same food samples [30,31]; this has also been documented in the case of hazelnut-based products [28]. Besides structural similarities, STE shares with AFB1 hepatotoxic and nephrotoxic effects, inducing IARC to classify them as a possible human carcinogen (group 2B) [32].

**Figure 3:**
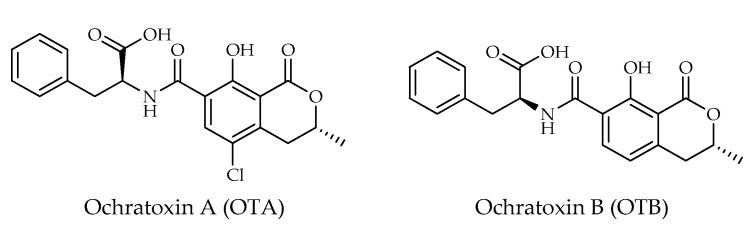
Chemical structures of ochratoxins (OTs) detected in hazelnuts.

As can be inferred from the existing literature, ochratoxin A (OTA) seems to be greatly diffused in hazelnuts [10,12,22,33], and as a contaminant of hazelnut-based food [28]. Moreover, the presence in hazelnuts of the dechloro analog of OTA, namely ochratoxin B (OTB), has been also reported [12,16]. Ochratoxins are mostly known as secondary metabolites of several *Aspergillus* and *Penicillium* spp. [34]. Biosynthetically, these mycotoxins are pentaketides derived from the dihydrocoumarin family coupled to phenylalanine (Figure 3). OTA is regarded as the most toxic member of ochratoxins, having been shown to be nephrotoxic, hepatotoxic, teratogenic, and immunotoxic to several species of animals. It has also been proven to be carcinogenic in kidney and liver, and has been classified as a group 2B human carcinogen by the IARC and World Health Organization (WHO) [35].

Most of the published data on ochratoxins, other than OTA, describe OTB toxicity. In fact, OTB was investigated for its nephrotoxic, hepatotoxic and immunotoxic effects [34].

**Figure 4:**
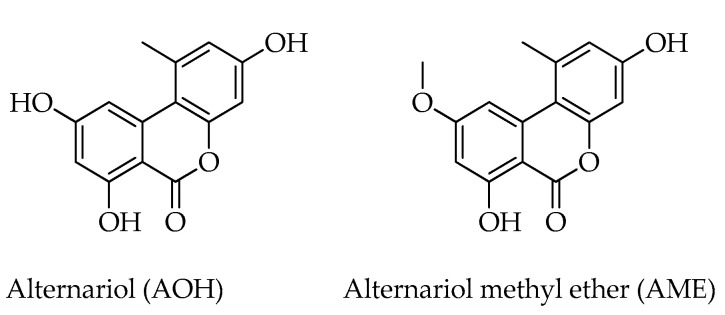
Chemical structures of resorcylic acid lactones detected in hazelnuts.

Commonly produced by *Alternaria* fungi, alternariol (AOH) and alternariol methyl ether (AME) were first identified in hazelnut samples by Varga et al. [12]. These mycotoxins belong to the group of resorcylic acid lactones which are characterized by the presence of a dibenzo-α-pyrone moiety (Figure 4). Even if no specific regulations in food and feed exist, AOH and AME are considered as emerging toxins because of the increasing evidence of their occurrence and toxicological properties [36]. To date, AOH has been reported to be cytotoxic, dermally toxic, and potentially carcinogenic. Moreover, various in vitro experiments and a few in vivo investigations were conducted to evaluate the genotoxicity of AOH [37].

**Figure 5:**
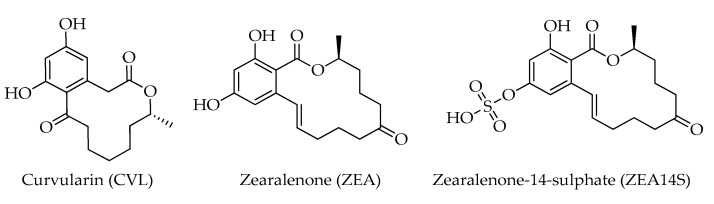
Chemical structures of macrolides detected in hazelnuts.

The 14-membered macrolide zearalenone (ZEA), also known as F-2 toxin, and zearalenone-14-sulphate (ZEA14S), are mainly produced by fungi of the genus *Fusarium* [38]. ZEA has immunotoxic, hepatotoxic, and xenogenic effects, and its activity in living organisms depends on the immune status of the organism and on the reproductive system state, due to the strong estrogenic and anabolic effects which have been reported [39]. These mycotoxins share the chemical structure of a macrocyclic β-resorcylic acid lactone with curvularin (CVL), another mycotoxin isolated from contaminated hazelnuts [12] (Figure 5).

**Figure 6:**
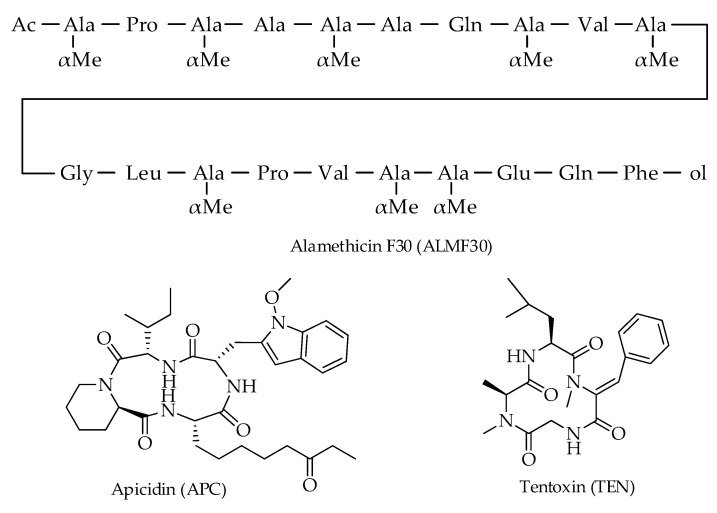
Chemical structures of amino acid and peptide derivatives detected in hazelnuts.

Apicidin (APC), alamethicin F30 (ALMF30), and tentoxin (TEN) are amino acid and peptide derivatives detected in hazelnuts [12,16]. APC and TEN are cyclic tetrapeptides, while ALMF30 is a 20-residue polypeptide belonging to the so-called peptaibiotics [40] (Figure 6).

**Figure 7:**
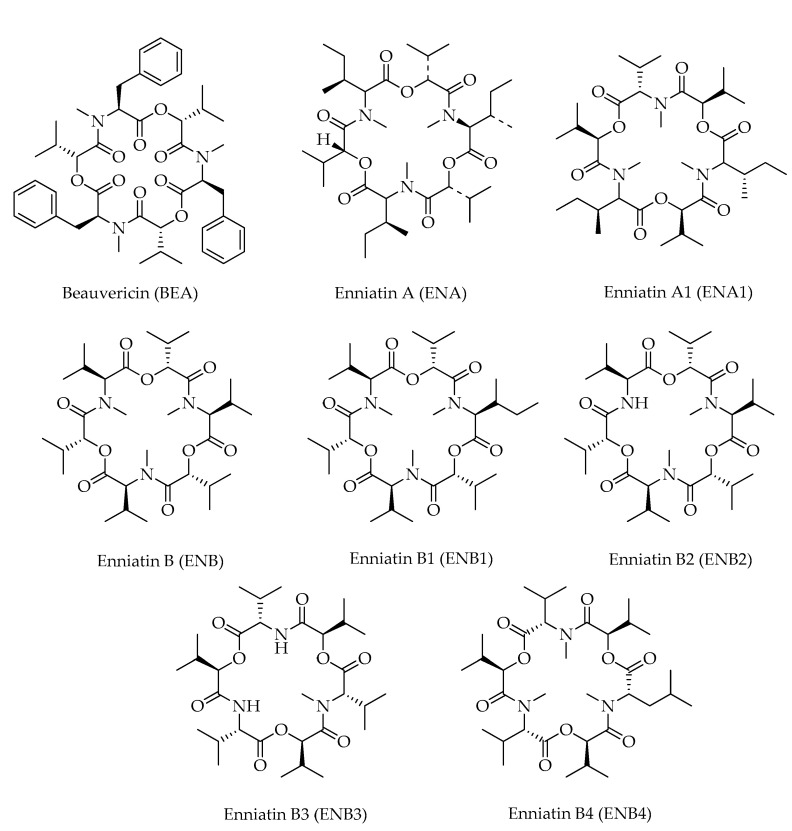
Chemical structures of cyclodepsipeptides detected in hazelnuts.

The class of cyclodepsipeptides includes N-methylated cyclic hexadepsipeptides, consisting of three residues of hydroxy acids (i.e., 2-hydroxyisovaleric acids) alternating with three N-methyl-L-amino acids, generally valine, leucine, and isoleucine. Peptide bonds and intramolecular ester bonds link the subunits to form an 18-membered ring. During the last few years, three papers have reported on the occurrence of depsipeptides in hazelnuts [12,16,21], including seven enniatins (ENs) and beauvericin (BEA) (Table 1). The latter presents three 2-hydroxyisovaleryl residues alternated to three N-methyl-phenylalanyl groups [41] (Figure 7). Again, these products are mainly known from *Fusaria* [42].

Members of this class are considered as emerging mycotoxins because mixtures or individual compounds have been shown to possess substantial in vitro cytotoxicity against different cell lines [41,43]. 

**Figure 8:**
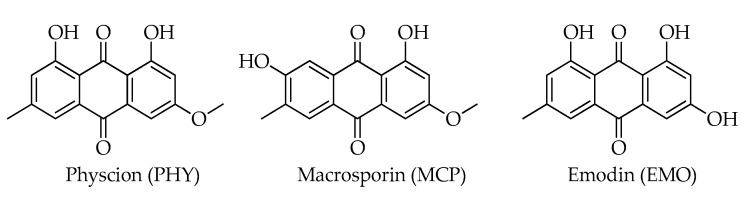
Chemical structures of anthraquinones detected in hazelnuts.

Three anthraquinones were detected in Turkish hazelnut samples by Varga et al. [12], namely physcion (= parietin, PHY), macrosporin (MCP), and emodin (EMO) (Figure 8). Anthraquinones are a valuable class of natural and synthetic compounds with a broad pharmacological function, including anti-bacterial, antioxidant, anti-tumor, and other activities, which are produced by many fungal species [44,45]. A growing number of toxicological data highlight the potential toxicity of compounds belonging this class [46].

**Figure 9:**
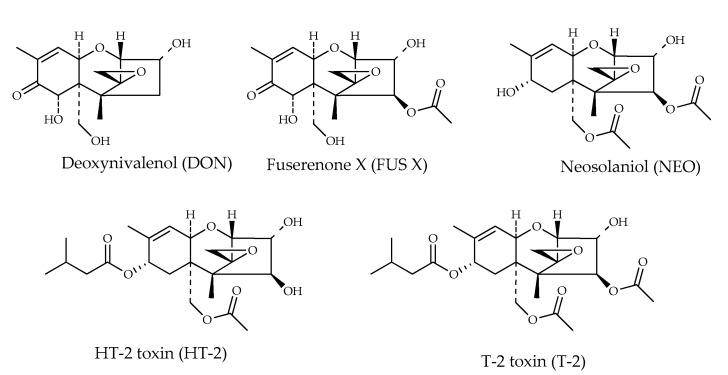
Chemical structures of trichothecenes detected in hazelnuts.

Several trichothecenes were identified from hazelnuts samples [12,19] and from hazelnut-based food [28]. Trichothecenes are a large family of sesquiterpenoids with the common core chemical structure consisting of a cyclohexene fused to a tetrahydropyran, which is bridged by a two-carbon chain forming a cyclopentyl moiety. A 12,13-epoxy ring completes this core (Figure 9). These fungal secondary metabolites are of major food safety concern because of the harmful effects that result from acute and chronic exposure [47,48]. They are produced by several fungi, including *Fusarium*, *Trichothecium*, *Trichoderma*, *Myrothecium*, and *Stachybotrys*, and they have an ample spectrum of toxicity for humans and animals [49].

**Figure 10:**
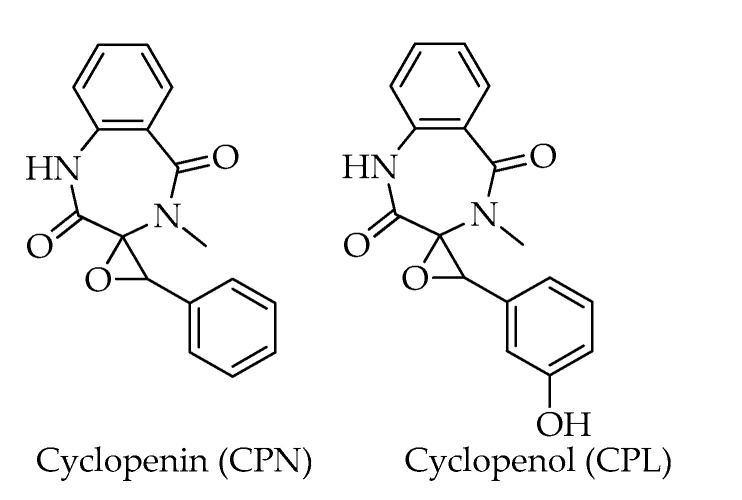
Chemical structures of benzodiazepine alkaloids detected in hazelnuts.

Cyclopenin (CPN) is a benzodiazepine alkaloid deriving from the cyclization of the dipeptide of anthranilic acid and phenylalanine [50]. In the biosynthesis, CPN serves as precursor of cyclopenol (CPL), which explains the frequent co-occurrence of these toxic fungal metabolites. Both these benzodiazepine alkaloids were identified in commercial samples of hazelnuts by Spadaro et al. [20] (Figure 10).

**Figure 11:**
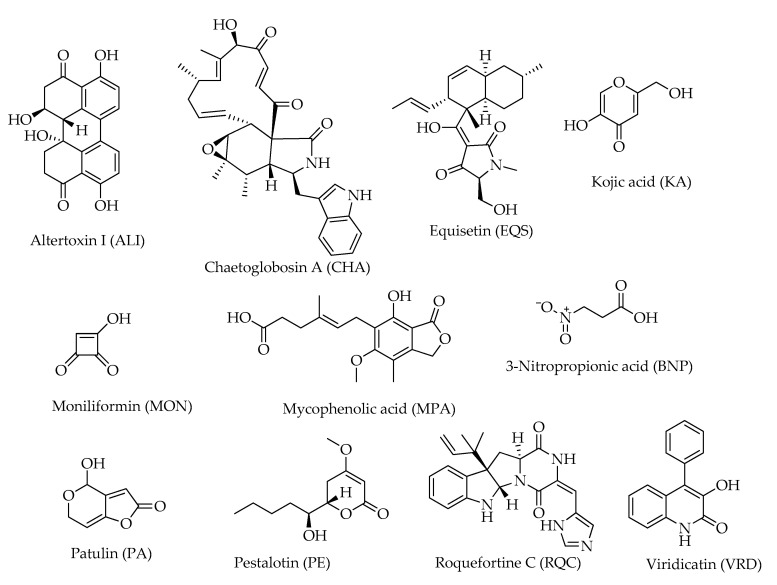
Structures of compounds from the group “miscellaneous” detected in hazelnuts.

Other mycotoxins detected in hazelnuts present heterogeneous structures (Figure 11). This is the case of patulin (PA), a polyketide lactone primarily produced by *Penicillium*, *Aspergillus*, and *Byssochlamys* spp., which is considered a serious health concern and an economic threat [51]. As the subject of a huge investigational activity, patulin content has also been determined in hazelnuts as function of fungal infections based on a relationship with aflatoxin and ergosterol concentrations in different categories of samples [11].

Mycophenolic acid (MPA) is a phenyl-terpenoid secondary metabolite produced by several species of *Penicillium* [52] showing antiviral, antibacterial, antitumoral, antifungal, and immunosuppressive activities [53]. It has been detected in hazelnuts in a couple of studies [12,20]; one of them [12] also reported the indole alkaloid roquefortine C, another mycotoxin essentially produced by the *Penicillium* species.

## 3. Analytical Methods for the Determination of Mycotoxins in Hazelnuts 

A number of methods have been developed for the identification and accurate quantification of single or chemically related mycotoxins in food samples [54]. Table 2 summarizes the analytical strategies employed for the detection of mycotoxins in real hazelnut samples. Many analytical methods have focused on the qualitative and quantitative determination of AFB1 and total aflatoxins, also prompted by the fact that these are the only mycotoxins included in the European regulation for hazelnuts [8,55,56]. However, different classes of mycotoxins could be found to co-occur in hazelnuts, with possible synergistic effects [57]. This is quite understandable, considering that many fungal species, which are reported as producers of toxic secondary metabolites belonging to different classes of natural compounds, can be concurrently isolated from hazelnuts, as is examined in more detail in Section 4.

The possible co-occurrence of different mycotoxins highlights that more information is needed on other fungal contaminants in hazelnuts, and stresses the importance of developing multi-mycotoxin approaches instead of single analyte methods, to monitor a higher number of compounds. 

The analysis of mycotoxins in hazelnuts is a challenging task, due to the complexity of the sample (i.e., high fat content) along with the low concentrations at which these contaminants are usually present. To cover the broad spectrum of mycotoxins, different analytical methods are often employed. 

Firstly, sample preparation, determinations, and analytical performance criteria must be coherent in order to obtain comparable data. In fact, the use of validated analytical methods is essential to ensure that the results of surveys provide a reliable content assessment. Based on the guidelines in the EU Commission Decision [58], the analytic methods with similar validation parameters, such as the limit of detection (LOD), limit of quantification (LOQ), linearity (r^2^), range of matrix effects, recovery, and relative standard deviation, are used for estimating the mycotoxin contamination levels. 

In general, conventional analytical methods, including TLC, LC-fluorescence, HPLC-UV, and ELISA are employed for the single or group target determination of mycotoxins, while LC-MS methods are preferred for multiclass analyses. 

Some LC-MS/MS methods for the simultaneous determination of toxic fungal metabolites in hazelnuts have been optimized and validated [12,20,33,59,60,61]. These methods include a first step of sample treatment based on solid–liquid extraction with an organic solvent. Although LC-MS has multi-analyte capabilities, the choice of extraction solvents and sample preparation may not be suitable for certain mycotoxins due to the high chemical diversity (Table 1). It was demonstrated that for the extraction of multiple contaminants in different food and feed matrices, a mixture of acidified water with organic solvents, such as methanol, acetonitrile, and acetone, is the most suitable system [62]. In general, a second step involves a clean-up using combinations of MgSO_4_ and different sorbents such as florisil, carbon black, C18, or primary and secondary amines, to remove interfering substances. For example, the procedure employed by Škrbić et al. [33] is based on the simultaneous extraction of selected mycotoxins from hazelnuts and other nuts with a mixture of acetonitrile/water/acetic acid (79:20:1, *v/v/v*), and defatting the obtained extract with hexane in order to remove the lipids. However, it was proven that the common cleaning-up decreases the recovery of mycotoxins [63]; hence, alternative cleaning-up methods, such as dispersive liquid–liquid microextraction (DLLME), have been employed for the analysis of mycotoxins [63]. DLLME is a three phase system constituting the extraction solvent, the dispersive solvent, and the aqueous phase. A suitable mixture of an organic extraction solvent (usually with a density higher than water) and a disperser solvent (miscible with the extraction solvent and with water) is rapidly injected into the aqueous phase, resulting in the formation of a stable emulsion. Centrifugation allows for phase separation, and the organic phase containing the analytes is subsequently analyzed using the chosen techniques [63,64,65]. Arroyo-Manzanares et al. [60] developed a multiclass method based on DLLME for the determination of 14 mycotoxins in different nuts and seeds, including hazelnuts. Nevertheless, every clean-up step is cost/time consuming and limits the number of analytes, as some of the target substances might not be amenable to the chosen procedure. Varga et al. [12] developed an UHPLC-MS/MS method, including a single extraction step and direct injection of the diluted raw extract into the instrument without any sample clean-up. This method allowed for the determination of several mycotoxins in different nut samples, including hazelnuts (Table 2).

As can be deduced from the above discussion, the choice of an appropriate multi-target methods for the quantification and determination of mycotoxins is essential for researchers involved in the study of toxic fungal metabolites in hazelnuts.

## 4. Mycotoxins in Hazelnuts and Fungal Infections

Kernel contamination by mycotoxins may derive from fungal infections occurring during fruit development, or in postharvest. In the field, the symptoms of fruit rot are various, in that they may involve the whole fruit and be visible externally, or they may specifically affect the kernel and be hidden by the shell. A list of fungi known as disease agents of hazelnut fruits is provided in Table 3. However, most frequently, the observed damage cannot be referred to a specific agent; rather, it results from overlapping infections by multiple species. On the other hand, the infectious capacity by several species is variable in space and time, with reference to the point of entry and the phenological stage of fruit development. In this respect, the incidence of *Diaporthe* spp. was found to be higher at the full ripening stage than in early ripening, and higher in defective than in healthy kernels. A similar pattern also characterized *Botryosphaeria*; however, the incidence of *Diaporthe* was positively correlated with both hidden and visible defects, while *Botryosphaeria* was essentially found in nuts with hidden defects [66]. The simultaneous occurrence in this study of *Diaporthe* and *Aspergillus* emphasizes the need to assess the outcome of their interaction, in terms of both kernel damage and the effects on mycotoxin production and accumulation. Due to the major concern for the accumulation of aflatoxins, some investigations have been carried out with special reference to *Aspergillus* spp., essentially species in the section *Flavi*. Indeed, their incidence may be quite remarkable in some environmental contexts, and is reported to increase throughout the season until the harvesting time [67].

The above data refer to fungal infections occurring in the field. Indeed, the issue of the fungal infestation of hazelnuts during storage and commercialization is basically different in its assumptions, considering that any saprophytic microbe may be able to contaminate the nuts at this stage, and to unpredictably contribute to mycotoxin accumulation.

A multitude of studies/investigations have been carried out concerning the fungal contamination of hazelnuts from commerce, particularly in Western Asian countries. In an investigation carried out in Saudi Arabia, 12 genera and 23 species were isolated, including a varied assortment of *Aspergillus* and *Penicillium* spp., which by far represents the most frequent contaminants at the marketing stage [87]. Isolates of *Aspergillus* (including *A. flavus*), *Penicillium, Rhizopus, Fusarium, Geotrichum, Syncephalastrum*, and *Cladosporium* were recovered in a Turkish survey [88]. Three studies carried out in Iran disclosed a broad set of fungal contaminants, consisting of *Alternaria, Aspergillus, Cladosporium, Drechslera, Fusarium, Mucor, Paecilomyces, Penicillium, Rhizopus, Scopulariopsis*, and *Trichothecium* [89,90,91]. *Aspergillus flavus, Aspergillus niger, Penicillium italicum*, and other *Penicillium* and *Cladosporium* spp. resulted in an investigation carried out in Iraq [10]. In another study carried out in Egypt, a total of 37 species were identified, including *Alternaria atra* (=*Ulocladium atrum*), *A. alternata, Chaetomium globosum, Cladosporium herbarum, Cladosporium cladosporioides, Cladosporium macrocarpum, Curvularia lunata* (=*Cochliobolus lunatus*), *Mucor circinelloides, Mucor hiemalis, Rhizomucor pusillus, Sarocladium* (=*Acremonium*) *strictum*, *Scopulariopsis brevicaulis, Talaromyces funiculosus, Talaromyces variabile, Trichocladium griseum* (=*Humicola grisea*), and *Trichothecium roseum*, six species of *Penicillium* and 15 species of *Aspergillus* [14]. An investigation carried out in Lithuania reported the occurrence of *A. niger*, *Aspergillus fumigatus, A. versicolor, Fusarium chlamydosporum* (=*F. sporotrichioides), Rhizopus stolonifer, Penicillium chrysogenum*, and other *Penicillium* spp. [92].

A couple of studies reported on fungi occurring in roasted hazelnuts, indicating that contamination with these fungi may be independent of the conditions of the product at harvest, and that rather, it can start during processing and marketing. Particularly, the species *T. roseum, Aspergillus glaucus, A. flavus,* and *A. niger*, as well as other *Aspergillus, Penicillium, Fusarium, Alternaria, Mucor*, and *Rhizopus* spp. were recovered in a Spanish study [93], while *Aspergillus* spp., mostly belonging to the sections *Flavi* and *Nigri*, were found to be quite abundant in an investigation carried out in Algeria, representing as much as 66% of the total fungal contaminants; however, just about half of the members of the section *Flavi* were found to be aflatoxigenic, essentially producing AFB1 [94]. Finally, as has occurred for mycotoxin analysis [95], other studies have been published that present collective data concerning other kind of nuts too, hence not allowing for an inference of specific associations with hazelnuts [15,96].

Most frequently, *Aspergillus* spp. are prevalent in these studies. However, the mycotoxin pattern remarkably varies among the species in this genus, calling for more circumstantial studies to further analyze the real taxonomic assortments involved in hazelnut contamination. In a recent study carried out in Iran, *Aspergillus* isolates from hazelnuts were identified to belong to 13 species grouped in 5 sections and 9 series based on sequencing of calmodulin and β-tubulin genes. Particularly, these are species from the section *Flavi*, including *A. caelatus* (series *Kitamyces*), *A. nomius* (series *Nomiarum*), *A. flavus, A. parasiticus*, and *A. arachidicola* (series *Flavi*); from the section *Nidulantes*, including *A. quadrilineatus* (series *Nidulantes*), *A. unguis* (series *Unguium*), and *A. spelunceus* (series *Speluncei*); from the section *Circumdati*, including *A. ochraceus* and *A. westerdijkiae* (series *Circumdati*); *A. pseudoglaucus* from the section *Aspergillus* (series *Rubri*); and *A. taichungensis* from section *Candidi* (series *Candidi*) [97,98]. Some of these species might be agents of contamination with additional mycotoxins, such as *A. pseudoglaucus*, which is known to produce echinulins [99]. In the above-mentioned Egyptian study, as many as 15 species were identified based on morphology, including *A. flavus, A. niger, A. ochraceus* (=*A. alutaceus*), *A. candidus, A. fumigatus, A. parasiticus, A. sydowii, A. tamari, A. terreus, A. versicolor, A. nidulans, A. amstelodami, A. chevalieri, A. rubrum*, and *A. rugulosus* [14]. Moreover, this study also reported on the occurrence of a varied assortment of *Penicillium* spp., including *P. chrysogenum, P. citrinum, P. corylophilum, P. cyclopium, P. janthinellum*, and *P. oxalicum*, which represents the second most frequent genus.

Although limited and somehow approximate, the available data are indicative that these fungi are reported as possible producers of about one half of the compounds listed in Table 1, namely aflatoxins, anthraquinones, cyclopenins, curvularin, ochratoxins, sterigmatocystins, chaetoglobosins, kojic acid, mycophenolic acid, patulin, and viridicatin [100,101]. However, their biosynthetic capacities go well beyond this limited number, and it should be taken into consideration that any mycotoxin that is known as a product of the species of these genera can potentially contaminate the kernels and the derivatives used in the food industry.

As for the other genera, an outstanding position pertains to *Diaporthe* (= *Phomopsis*), considering the high number of species reported in association with hazelnuts, and the remarkable biosynthetic capacities that exteriorize in a long series of secondary metabolites that are so far reported from these fungi [102,103]. With reference to the mycotoxins listed in Table 1, production is to be mentioned of 3-nitropropionic acid by a pathogenic strain of *D. gulyae* on sunflower [104], alternariol and alternariol methyl ether by an endophytic strain of an unidentified species [105], and emodin by an endophytic strain of *D. lithocarpi* [106]. Moreover, the sterigmatocystin analogues secosterigmatocystin and dihydrosterigmatocystin have been extracted from an endophytic strain of *D. amygdali* [107]. However, more products of these fungi have been reported for their toxic effects, which should be considered as possible contaminants of hazelnuts; this is the case for phomoxanthone, pinselin, and other xanthones, as well as several benzofuranones, quinones, and alkaloids [103].

## 5. Control Strategies 

Several pre- and post-harvest operations of hazelnuts and other food products can help with controlling the fungal contamination, and also significantly reduce the quantity of mycotoxins in them [108,109]. Chemical control is a successful strategy in crop protection for reducing mycotoxigenic fungi in the field, but it is associated with undesirable effects. The application of appropriate storage conditions (e.g., the use of hermetic containers, temperature and humidity control, and ventilation) is an essential post-harvest strategy to avoid fungal growth and mycotoxins accumulation [110,111]. Moreover, the additional processing of these commodities may be associated with secure and safe consumption.

Several decontamination strategies, including physical (e.g., cleaning, thermal inactivation, irradiation with UV or gamma rays, and cooling), chemical (e.g., treatments with hydrogen peroxide, acids or bases, or enzymes) or biological (e.g., atoxigenic microbes) tools, have been tested against fungi and their mycotoxins. However, these methods may cause undesirable effects on the sensory, nutritional and functional properties of foods [6].

A promising non-thermal alternative for reducing the fungal load is cold atmospheric plasma, which enables a microbial multi-target inactivation on food surfaces, covering an ample range of microorganisms, including bacteria and fungi [112,113]. Plasma is considered as the fourth state of matter obtained by energetic species formed from the collisions of energetic electrons with heavy particles (e.g., atoms, molecules, and ions). Cold plasma generated under atmospheric pressure produces antimicrobial effects at temperature below 40 °C. Dasan et al. focused on the effect of process parameters on the inactivation efficiency of cold atmospheric plasma on aflatoxigenic *Aspergillus* spp. spores in hazelnuts [114,115]. To achieve this goal, hazelnuts were artificially contaminated with *A. flavus* and *A. parasiticus,* then treated with dry air or cold plasma. On an experimental scale, these studies showed that cold atmospheric plasma is an efficient post-harvest sanitation system, affording a reasonable reduction in contamination by *Aspergillus* spp.

Cold atmospheric plasma has the potential for aflatoxin detoxification in food, also because it preserves the organoleptic properties. Siciliano et al. [116] used cold atmospheric plasma to detoxify hazelnuts from aflatoxins, investigating the effect of different gases (N_2_, 0.1% O_2_ and 1% O_2_, or 21% O_2_), power (400, 700, 1000, or 1150 W) and exposure time (1, 2, 4, or 12 min) to optimize the method. A reduction in the concentration of total aflatoxins and AFB1 in hazelnuts of over 70% was obtained. This result was also confirmed by Sen et al. [117], who observed that cold atmospheric pressure and low-pressure plasmas are more effective than gamma irradiation for the reduction in AFB1 and total aflatoxins in hazelnuts. Furthermore, sensory evaluation tests showed that hazelnuts maintain optimal attributes after these treatments.

Even if the mechanism of degradation or resistance of mycotoxins is not fully understood, it is thought that a primary role is played by the mycotoxin structure [113]. In fact, it has been observed that the sensitivities of AFB1 and AFG1 to cold atmospheric plasma are higher than AFB2 and AFG2. The reason was attributed to the possible destruction of the C8–C9 double bond (olefinic site) on the furan ring, which is responsible for the toxicities of AFB1 and AFG1; whereas this double bond is not present in AFB2 and AFG2 [116]. These treatments cause the opening of the terminal furan ring in the double bond, leading to the formation of organic acids, aldehydes, ketones, and other degradation products [118].

## 6. Conclusions

In the present review paper, the available data concerning the literature on mycotoxins detected in hazelnuts were examined. A high variety of mycotoxins with different chemical properties and toxicities have been detected in the hazelnut samples. These toxic fungal metabolites can be classified at least in 10 groups (i.e., aflatoxins, amino acid and peptide derivatives, anthraquinones, benzodiazepine alkaloids, cyclodepsipeptides, macrolides, ochratoxins, resorcylic acid lactones, sterigmatocystins, and trichothecenes). 

Mycotoxins and fungal producers represent a great public health issue. Hence, further investigations should also be carried out to increase the available data concerning conditions that are conducive to the development of mycotoxigenic fungi in the field, particularly with reference to the possible effects deriving from interactions with other components of the hazelnut microbiome [119,120].

Evidence from investigations carried out by several laboratories and research groups worldwide supports concern for the contamination of hazelnuts by mycotoxins. Indeed, the increasing number of fungal secondary metabolites identified in kernels and that are known to possibly induce a range of toxic effects on human health, calls for a revision of the analytical procedures that are currently limited to aflatoxins, at least in the European Union.

## Figures and Tables

**Table 1:** Mycotoxins detected in hazelnuts.

Mycotoxin	Formula	Nominal Mass (U)	Reference
**Aflatoxins**			
Aflatoxin B1 (AFB1)	C_17_H_12_O_6_	312	[8,9,10,11,12,13,14,15,16,17,18]
Aflatoxin B2 (AFB2)	C_17_H_14_O_6_	314	[8,9,10,11,12,13,14,16,17,18]
Aflatoxin G1 (AFG1)	C_17_H_12_O_7_	328	[8,9,10,11,12,13,14,17,18]
Aflatoxin G2 (AFG2)	C_17_H_14_O_7_	330	[9,10,11,12,13,14,17,18,19]
**Amino acid derivatives**			
Alamethicin F30 (ALMF30)	C_92_H_150_N_22_O_25_	1964	[12]
Apicidin (APC)	C_34_H_49_N_5_O_6_	624	[12]
Tentoxin (TEN)	C_22_H_30_N_4_O_4_	414	[12,16]
**Anthraquinones**			
Emodin (EMO)	C_15_H_10_O_5_	270	[12]
Macrosporin (MCP)	C_16_H_12_O_5_	284	[12]
Physcion (= parietin) (PHY)	C_16_H_12_O_5_	284	[12]
**Benzodiazepine alkaloids**			
Cyclopenin (CPN)	C_17_H_14_N_2_O_3_	294	[20]
Cyclopenol (CPL)	C_17_H_14_N_2_O_4_	310	[20]
**Cyclodepsipeptides**			
Beauvericin (BEA)	C_45_H_57_N_3_O_9_	784	[12,21]
Enniatin A (ENA)	C_36_H_63_N_3_O_9_	682	[12,16,21]
Enniatin A1 (ENA1)	C_35_H_61_N_3_O_9_	668	[12,16,21]
Enniatin B (ENB)	C_33_H_57_N_3_O_9_	640	[12,16,21]
Enniatin B1 (ENB1)	C_34_H_59_N_3_O_9_	654	[16,21]
Enniatin B2 (ENB2)	C_32_H_55_N_3_O_9_	626	[12]
Enniatin B3 (ENB3)	C_31_H_53_N_3_O_9_	612	[12]
Enniatin B4 (ENB4)	C_34_H_59_N_3_O_9_	654	[12]
**Macrolides**			
Curvularin (CVL)	C_16_H_20_O_5_	292	[12]
Zearalenone (ZEA)	C_18_H_22_O_5_	318	[12]
Zearalenone-14-sulphate (ZEA14S)	C_18_H_22_O_8_S	398	[12]
**Ochratoxins**			
Ochratoxin A (OTA)	C_20_H_18_ClNO_6_	404	[10,12,22]
Ochratoxin B (OTB)	C_20_H_19_NO_6_	369	[12,16]
**Resorcylic acid lactones**			
Alternariol (AOH)	C_14_H_10_O_5_	258	[12,16]
Alternariol methyl ether (AME)	C_15_H_12_O_5_	272	[12,16]
**Sterigmatocystins**			
3-O-Methylsterigmatocystin (OMST)	C_19_H_14_O_6_	338	[12]
Sterigmatocystin (STE)	C_18_H_12_O_6_	324	[12]
**Trichothecenes**			
Deoxynivalenol (DON)	C_15_H_20_O_6_	296	[19]
Fuserenone X (FUS X)	C_17_H_22_O_8_	354	[19]
HT-2 toxin (HT-2)	C_22_H_32_O_8_	424	[12]
Neosolaniol (NEO)	C_19_H_26_O_8_	382	[19]
T-2 toxin (T-2)	C_24_H_34_O_9_	467	[12]
**Miscellaneous**			
Altertoxin I (ALI)	C_20_H_16_O_6_	352	[12]
Chaetoglobosin A (CHA)	C_32_H_36_N_2_O_5_	529	[20]
Equisetin (EQS)	C_22_H_31_NO_4_	373	[12]
Kojic acid (KA)	C_6_H_6_O_4_	142	[12]
Moniliformin (MON)	C_4_H_2_O_3_	98	[12]
Mycophenolic acid (MPA)	C_17_H_20_O_6_	320	[12,20]
3-Nitropropionic acid (BNP)	C_3_H_5_NO_4_	119	[12]
Patulin (PA)	C_7_H_6_O_4_	154	[11]
Pestalotin (PE)	C_11_H_18_O_4_	214	[12]
Roquefortine C (ROQC)	C_22_H_23_N_5_O_2_	389	[20]
Viridicatin (VRD)	C_15_H_11_NO_2_	237	[12]

**Table 2:** Analytical strategies employed to determine mycotoxins in real hazelnut samples.

Type of Sample	Mycotoxins	Samples	Sample Preparation	Detection	Levels (µg kg^−1^)	Ref.
Hazelnuts	AFB1	35	Ultrasound extraction with ACN:H_2_O (8:2, *v/v*), cleaning up with a Carbograph-4 SPE cartridge eluted with CH_2_Cl_2_:MeOH:acetic acid (88:10:2, *v/v/v*)	LC/ESI-MS/MS Mobile phase: (A) ACN:H_2_O (95:5, *v/v*); (B) H_2_O	not detected (n.d.)–0.9	[8]
	AFB2				n.d.–<LOQ	
	AFG1				n.d.–0.1	
Hazelnut paste	AFB1	5	Extraction with MeOH:H_2_O (8:2, *v/v*) and *n*-hexane, cleaning up with immunoaffinity columns (IAC) eluted with MeOH	HPLC-FLD Mobile phase: ACN:MeOH:H_2_O (20:20:60, *v/v/v*)	0.45–3.61	[9]
	AFB2				<LOQ–0.55	
	AFG1				n.d.–1.84	
	AFG2				<LOQ–0.30	
Hazelnuts without shell	AFB1	32			0.20	
	AFG1				031	
Roasted hazelnuts	AFB1	9			3.45	
	AFB2				1.16	
	AFG1				0.16	
	AFG2				1.82	
Hazelnuts	Total AFs	-	Extraction with 70% MeOH and filtration	Commercially available kit based on CD-ELISA	10.3	[10]
	OTA				1.5	
Sound hazelnuts	AFB1	5	AFs determination: extraction in MeOH:H_2_O (8:2, *v/v*), cleaning up with immunoaffinity assays. PA determination: extraction with ethyl acetate and filtration, subsequent extraction with 3% sodium carbonate solution, acidification of the organic phase	AFs determination: HPLC-FLD Mobile phase: H_2_O:ACN:MeOH (6:2:3, *v/v/v*). PA determination: HPLC-DAD: Mobile phase: H_2_O:ACN (1:9, *v/v*)	0.4–0.9	[11]
Moldy hazelnuts	AFB1				510–246	
	AFB2				4.4–1.6	
	AFG1				205–98.7	
	AFG2				1.3–4.0	
	PA				65.8–25.6	
Hidden moldy hazelnuts	AFB1				422–141	
	AFB2				0.8–2.0	
	AFG1				78.6–96.4	
	AFG2				0.5–2.1	
	PA				67.6–16.6	
Hazelnuts	AFB1	22	Extraction with ACN:H_2_O:Acetic acid (79:20:1, *v/v/v*), dilution of the extract with ACN:H_2_O:acetic acid (79:20:1, *v/v/v*)	UHPLC-MS/MS Mobile phase: (A) MeOH:H_2_O:Acetic acid (10:89:1, *v/v/v*) (B) MeOH:H_2_O:Acetic acid (97:2:1, *v/v/v*)	7.4	[12]
	AFB2				5.5	
	AFG1				16	
	AFG2				5.5	
	ALMF30				110	
	AOH				78	
	AME				59	
	ALI				7.0	
	APC				3.4	
	BEA				2.4	
	CVL				19	
	EMO				5.5	
	ENA				28	
	ENA1				140	
	ENB				37	
	ENB2				3.0	
	ENB3				0.06	
	ENB4				22	
	EQS				110	
	HT-2				39	
	KA				1100	
	MCP				280	
	OMST				1.7	
	MPA				700	
	BNP				440	
	OTA				220	
	OTB				6.9	
	PE				3.1	
	PHY				700	
	STE				2.3	
	T-2				32	
	TEN				5.4	
	VRD				5.7	
	ZEA				7.6	
	ZEA14S				3.9	
Hazelnuts	AFB1	42	Extraction with MeOH:H_2_O, cleaning up with immunoaffinity columns	HPLC-FLD	1.37	[13]
	Total AFs				4.11	
Hazelnuts	AFB1	20	Soxhlet extraction with *n*-hexane, subsequent extraction with CHCl_3_, cleaning up with silica gel columns	TLC Mobile phase: MeOH:CHCl_3_ (3:97, *v/v*)	25–175	[14]
	AFB2				25–175	
	AFG1				25–175	
	AFG2				25–175	
Hazelnuts	AFB1	28	Extraction with CH_2_Cl_2,_ cleaning up with columns eluted with CHCl_3_:acetone (90:10, *v/v*)	TLC Mobile phase: diethyl ether	34.4 ppb	[15]
Edible part of hazelnuts	AFB1	20	Extraction with acidified ACN, cleaning up with C18 sorbent	UPLC-MS/MS Mobile phase: (A) ACN (B) 0.5% formic acid in water with 10 mMol/L citric acid	-	[16]
	AFB2				-	
	ENA				1.00	
	ENA1				4.48	
	ENB				1.58	
	ENB1				1.04	
	Total Afs				< LOQ–2.10	
Raw hazelnuts	Total AFs	30	Neogen Veratox^®^	CD-ELISA	2.11–10.03	[17]
Roasted hazelnuts	Total AFs	50			0.1–4.04	
Inner membrane of hazelnuts	Total AFs	50			0.7–38.2	
Hazelnuts	AFs	43	Immunocompetition assay	ELISA	-	[18]
Hazelnuts	AFG2	7	QuEChERS extraction with acidified ACN, d-SPE cleaning up with C18 and Z-Sep+	HPLC-MS/MS Mobile phase: (A) H_2_O:MeOH:Acetic acid (94:5:1, *v/v/v*) (B) H_2_O:MeOH:Acetic acid (97:2:1, *v/v/v*)	2.6	[19]
	DON				56.01	
	FUS X				45.09	
	NEO				<LOQ	
Hazelnuts	CHA	13	Sequential extractions with solvents of different polarity	HPLC-MS/MS Mobile phase: (A) acidified H_2_O (B) ACN.	7.6–29.2	[20]
	CPN				1.32–1.37	
	CPL				11.02–21.45	
	MPA				2.7	
	ROQC				<LOQ	
Hazelnut fruit	ENA	4	Ultrasonic extraction with CAN, cleaning up with C18 columns. Ultrasonic extraction of the residues dissolved in ACN:MeOH (5:5, *v/v*)	LC-MS/MS Mobile phase (gradient elution): (A) MeOH (B) ACN	0.263	[21]
	ENA1				0.007	
	ENB				0.146	
Hazelnut shell	BEA				0.03	
	ENA				0.732	
	ENB				0.076	
	ENB1				0.417	
Hazelnuts	OTA	1	Extraction with MeOH:H_2_O (7:3, *v/v*)	Commercially available kit based on ELISA	-	[22]

**Table 3:** Fungal species reported as infecting hazelnut fruit.

Species	Country	References
*Alternaria alternata*	Chile	[68]
	Italy	[69]
*Alternaria arborescens*	Italy	[69]
*Alternaria* sp.	Georgia	[70]
	Nebraska (USA)	[71]
	Turkey	[72]
*Alternaria tenuissima*	Italy	[69]
*Aspergillus* sp.	Oregon (USA)	[73]
	Georgia	[70]
	Turkey	[66,72,74]
*Botryosphaeria* sp.	Turkey	[66,72]
*Botrytis cinerea*	Turkey	[75]
*Botrytis* sp.	Georgia	[70]
	Turkey	[72]
*Chrysonilia* sp.	Nebraska (USA)	[71]
*Ciboria (Monilia) coryli*	Poland	[76]
*Cladosporium* sp.	Georgia	[70]
	Nebraska, Oregon (USA)	[71,73]
	Turkey	[72,74]
*Colletotrichum acutatum*	Turkey	[75]
*Colletotrichum fioriniae*	Turkey	[77]
*Colletotrichum* sp.	Georgia	[70]
*Diaporthe arecae*	Turkey	[72]
*Diaporthe eres*	Georgia	[70]
	Turkey	[72]
*Diaporthe foeniculina*	Chile	[78]
*Diaporthe hongkongensis*	Turkey	[72]
*Diaporthe oculi*	Turkey	[72]
*Diaporthe pseudoculi*	Turkey	[72]
*Diaporthe rudis*	Oregon (USA)	[73]
*Diaporthe sojae*	Turkey	[72]
*Diaporthe* sp.	Chile	[68]
	Georgia	[70]
	Turkey	[66,72]
*Diaporthe unshiuensis*	Turkey	[72]
*Didymella corylicola*	Italy	[79]
*Diplodia* sp.	Oregon (USA)	[73]
*Eremothecium coryli*	Bulgaria	[80]
	Oregon (USA)	[81]
*Eremothecium cymbalariae*	Bulgaria	[82]
*Fusarium chlamydosporum* (= *F. sporotrichioides*)	Chile	[68]
*Fusarium culmorum*	Oregon (USA)	[73]
*Fusarium lateritium*	Italy	[83]
	Oregon (USA)	[73]
*Fusarium* sp.	Georgia	[70]
	Nebraska (USA)	[71]
	Turkey	[66,72]
*Fusarium tricinctum*	Italy	[84]
*Gnomoniopsis idaeicola*	Oregon (USA)	[73]
*Mucor* sp.	Turkey	[72]
*Neofusicoccum* sp.	Chile	[68]
*Paecilomyces* sp.	Nebraska (USA)	[71]
*Penicillium* sp.	Georgia	[70]
	Nebraska, Oregon (USA)	[71,73]
	Turkey	[66,72,74]
*Pestalotiopsis* sp.	Georgia	[70]
	Turkey	[72,85]
*Phoma* sp.	Georgia	[70]
*Ramularia* sp.	Oregon (USA)	[73,86]
*Rhizopus* sp.	Georgia	[70]
	Turkey	[72]
*Septoria* sp.	Georgia	[70]
*Sphaceloma* sp.	Georgia	[70]
*Trichoderma* sp.	Turkey	[72]
*Trichothecium roseum*	Turkey	[74]
*Trichothecium* sp.	Georgia	[70]
	Turkey	[72]

## Data Availability

Not applicable.
